# Investigating Sulforaphane’s anti-virulence and anti-quorum sensing properties against *Pseudomonas aeruginosa*

**DOI:** 10.3389/fphar.2024.1406653

**Published:** 2024-05-21

**Authors:** Mahmoud M. Bendary, Mohamed A. M. Ali, Alyaa S. Abdel Halim, Fehmi Boufahja, Anis Ahmad Chaudhary, Amr Elkelish, Rania H. M. Soliman, Wael A. H. Hegazy

**Affiliations:** ^1^ Department of Microbiology and Immunology, Faculty of Pharmacy, Port Said University, Port Said, Egypt; ^2^ Department of Biology, College of Science, Imam Mohammad Ibn Saud Islamic University (IMSIU), Riyadh, Saudi Arabia; ^3^ Department of Biochemistry, Faculty of Science, Ain Shams University, Cairo, Egypt; ^4^ Department of Botany and Microbiology, Faculty of Science, Suez Canal University, Ismailia, Egypt; ^5^ Department of Anatomy and Embryology, Faculty of Medicine, Zagazig University, Zagazig, Egypt; ^6^ Department of Microbiology and Immunology, Faculty of Pharmacy, Zagazig University, Zagazig, Egypt; ^7^ Pharmacy Program, Department of Pharmaceutical Sciences, Oman College of Health Sciences, Muscat, Oman

**Keywords:** Sulforaphane, *Pseudomonas aeruginosa*, quorum sensing, bacterial virulence, resistance to antibiotics

## Abstract

**Background:**

*P. aeruginosa*, a significant bacterium, can cause severe illness and resistance to antibiotics. Quorum sensing (QS) systems regulate virulence factors production. Targeting QS could reduce bacteria pathogenicity and prevent antibiotic resistance. Cruciferous vegetables contain sulforaphane, known for its anti-inflammatory, antioxidant, anticancer, and antimicrobial properties.

**Aim:**

We aimed to examine the inhibitory influences of sulforaphane, at a sub-inhibitory concentration (¼ minimum inhibitory concentration, MIC), on virulence and QS in *P. aeruginosa*.

**Materials and methods:**

The sulforaphane’s anti-virulence actions at sub-inhibitory concentrations were explored *in vitro* and *in vivo*. A sub-MIC concentration of sulforaphane was combined with anti-pseudomonal drugs, and the results of this combination were assessed. The virtual affinity of sulforaphane for the receptors of QS was studied, and its effect on the expression of QS genes was quantified.

**Results:**

Sulforaphane significantly decreased the biofilm formation, motility, ability to withstand oxidative stress, and the synthesis of virulence extracellular enzymes such as proteases, hemolysins, and elastase, as well as other virulence factors like pyocyanin. In addition, sulforaphane lessened the severity of *P. aeruginosa* infection in mice. Sulforaphane reduced the antipseudomonal antibiotics’ MICs when used together, resulting in synergistic effects. The observed anti-virulence impacts were attributed to the ability of sulforaphane to inhibit QS via suppressing the QS genes’ expression.

**Conclusion:**

Sulforaphane shows promise as a potent anti-virulence and anti-QS agent that can be used alongside conventional antimicrobials to manage severe infections effectively. Furthermore, this study paves the way for further investigation of sulforaphane and similar structures as pharmacophores for anti-QS candidates.

## 1 Introduction

*Pseudomonas aeruginosa* is a bacterium that belongs to the Gram-negative group. It takes advantage of weakened immune systems, particularly in those with conditions like cystic fibrosis or those who are hospitalized, to cause infections (Horcajada et al., 2019). The severity of infections produced by *P. aeruginosa* can vary in severity, ranging from moderate localized infections to severe fulminant infections and pneumonia (Rossolini and Mantengoli, 2005; Oliver et al., 2015). It is notorious for its capacity to develop resistance to various categories of antibiotics and antimicrobials, which exacerbates infections and complicates treatment (Parai et al., 2018; Pang et al., 2019). *P. aeruginosa* can create biofilms on surfaces that are either non-living or living, thereby offering a shield against the immune system of the host as well as the effects of antibiotics. Biofilms also have a role in the long-lasting nature of infections and the colonization of medical devices (Maurice et al., 2018). Further, the extensive spectrum of virulence factors utilized by *P. aeruginosa* highlights its flexibility and capacity to generate various infections in different host environments (Gellatly and Hancock, 2013; Nazeih et al., 2023). Virulence factors encompass the synthesis of exotoxins and extracellular enzymes that can affect the functioning of host cells and lead to tissue damage (Moradali et al., 2017). *P. aeruginosa* is renowned for its capability to synthesize a wide variety of enzymes, contributing significantly to its pathogenicity and adaptability. Among these enzymes, proteases play a significant function in the degradation of host tissues and immune evasion by cleaving various host proteins involved in the immune response (Carniol and Gilmore, 2004; Voynow et al., 2008). Elastases, another class of enzymes generated by *P. aeruginosa*, target elastin and other structural proteins, leading to injury to the host’s tissues and impairment of host defenses (Ohman et al., 1980; Tamura et al., 1992). Additionally, *P. aeruginosa* secretes hemolysins, which erythrocytes, releasing nutrients that promote bacterial growth and dissemination (Rossignol et al., 2008). Furthermore, *P. aeruginosa* produces pyocyanin, a blue-green pigment; this pigment acts as both a marker for virulence and a vital component in the development of the disease caused by this bacterium. Pyocyanin contributes to the bacterium’s resistance to oxidative stress and modulates the immune response of the host, thereby leading to tissue damage and inflammation (Jayaseelan et al., 2014; Alatraktchi et al., 2020). *P. aeruginosa* also utilizes secretion systems, such as the type III secretion system (T3SS) and type VI secretion system (T6SS), to transport toxins and other effector molecules directly into host cells. This mechanism aids in the bacterium’s survival and spread (Sadikot et al., 2005; Pena et al., 2019). Collectively, this arsenal of virulence factors plays a significant role in *P. aeruginosa’s* capacity to establish infections in a diverse array of host niches.

In addition, *P. aeruginosa* employs quorum sensing (QS) to regulate the virulence factors’ expression in response to the bacterial population’s density, which enables coordinated bacterial behavior and increases pathogenicity (Juhas et al., 2005; Khayat et al., 2022a; Ghaly et al., 2023). QS plays a significant role in the coordination of a variety of bacterial functions, including regulating virulence factor generation, forming biofilms, controlling motility, and influencing antibiotic resistance (Venturi, 2006; Skindersoe et al., 2008). Bacteria can utilize QS to perceive changes in population density and adapt their activity accordingly. This ability enables them to flourish and enhance their pathogenicity in various environmental conditions (Chen et al., 2011; Li and Tian, 2012). Directing efforts towards QS pathways provides a potential strategy for the treatment of bacterial infections and reduces their associated pathogenicity (Hentzer et al., 2003; Kipnis et al., 2006). By inhibiting QS signaling, it is feasible to interfere with the synchronized expression of virulence proteins, reducing bacterial pathogenicity without directly causing bacterial death (Rasmussen and Givskov, 2006). This method provides multiple benefits compared to conventional antibiotic treatment, such as less selective pressure for developing resistance and maintaining the beneficial microbiota (Elfaky et al., 2022b; Cavalu et al., 2022). Furthermore, directing efforts towards QS has the capacity to improve the effectiveness of current antibiotics by making bacteria more responsive to antimicrobial drugs (Thabit et al., 2022a; b). By interfering with the regulation of the generation of biofilm and the synthesis of virulence factors by bacteria, it is feasible to increase their susceptibility to the host’s immune system and antibiotic therapies, resulting in better clinical results (Rutherford and Bassler, 2012). Multiple natural (Aldawsari et al., 2021; Elfaky et al., 2022a; Khayat et al., 2022c; Khayat et al., 2023b; Koshak et al., 2023) and synthetic compounds (Almalki et al., 2022a; Almalki et al., 2022c; Alotaibi et al., 2023) have been tested to determine their effectiveness in suppressing bacterial QS and pathogenicity.

Sulforaphane, an isothiocyanate chemical that occurs naturally, can be found in cruciferous vegetables, including cabbage, broccoli, and kale (Yagishita et al., 2019). It has attracted interest due to its potential health advantages, including its antioxidant and anti-inflammatory characteristics and its capacity to stimulate detoxification enzymes (Fimognari and Hrelia, 2007; Guerrero-Beltrán et al., 2012; Briones-Herrera et al., 2018). Furthermore, sulforaphane has shown significant antibacterial effects against *Helicobacter pylori* (Fahey et al., 2002; Fahey et al., 2013) and other enteric bacteria such as *Escherichia coli* (Nowicki et al., 2019), and *Shigella sonnei* (Mazarakis et al., 2021), as well as cutaneous pathogenic bacteria, including *Staphylococcus aureus* and *Streptococcus pyogenes* (Ko et al., 2016; Deramaudt et al., 2020), and respiratory pathogens as *Haemophilus influenzae* (Mazarakis et al., 2021). Because of the abovementioned sulforaphane’s benefits to health, and owing to its safety at higher concentrations (Yagishita et al., 2019), it is used as a food supplement. The discovery of these data motivates us to explore the anti-virulence properties of sulforaphane further, indicating its potential as a supplementary therapy in combination with standard antibiotics for treating clinically significant pseudomonal infections.

## 2 Materials and methods

### 2.1 Materials

All of the media utilized were obtained from Oxoid Ltd (United Kingdom). All the chemicals utilized are of pharmaceutical grade. The compound L-sulforaphane, with the CAS Number 142825-10-3, was purchased from Cayman Chemical (Michigan, United States). The antimicrobial assessments were carried out against the common reference strain *P. aeruginosa* PAO1 (Nazeih et al., 2023).

### 2.2 Detection of sulforaphane minimum inhibitory concentration against *P. aeruginosa*

The MIC of sulforaphane was detected utilizing the broth microdilution method following the directives outlined by the Clinical Laboratory and Standards Institute (CLSI, 2015) (Khayat et al., 2022b). Briefly, two-fold serial dilutions of sulforaphane from 32 mg/mL to 0.25 μg/mL were prepared in Mueller–Hinton broth. One hundred µl aliquots were transferred into the microtiter plates to be mixed with an equal volume of bacterial suspensions with an approximate density of 1 × 10^6^ CFU/mL. Following an overnight incubation of the microtiter plate at 37°C, the wells were inspected for growth, and the MIC was identified as the lowest concentration that prevented visible bacterial growth.

### 2.3 Formation of biofilms

The crystal violet technique was utilized to assess the impact of sulforaphane on biofilm development at sub-MIC levels (Khayat et al., 2022a). In summary, PAO1 was cultured overnight at 37°C in Tryptone Soya Broth (TSB) and diluted to a concentration of 1 × 10^6^ CFU/ml. A volume of 0.1 mL of bacterial suspensions was put into microtiter plate wells with either TSB or TSB with ¼ MIC of sulforaphane. Following a 24-h incubation period at a temperature of 37°C, the free-floating bacteria were eliminated, and the wells were cleansed and treated with methanol (99%) for fixation. Subsequently, staining with crystal violet (CV) at a concentration of 1% was done. The surplus CV was eliminated, and the optical density was quantified at 590 nm after adding glacial acetic acid (33%) to dissolve the bound CV.

### 2.4 Protease assay

The study employed the skim milk agar technique (Khayat et al., 2022c) to assess the ability of sulforaphane to decrease PAO1 proteolytic activity. Supernatants obtained from PAO1 cultures grown overnight in Luria Broth (LB), both with and without sulforaphane at a concentration of ¼ of the MIC, were placed into preformed wells on plates of skim milk agar (5% concentration). After being incubated at a temperature of 37°C overnight, the clear zones that formed around the wells were detected.

### 2.5 Hemolysins assay

To estimate the anti-hemolytic influence of sulforaphane, the supernatants obtained from cultures that were treated with or without sulforaphane at a concentration of ¼ of the MIC were thoroughly mixed with fresh suspensions of rabbit blood at a concentration of 2%. The optical density at 540 nm was detected following centrifugation, after 2 h incubation at a temperature of 37°C. The blood suspensions were treated with sodium dodecyl sulfate (SDS) at a concentration of 0.1% (positive control), while the untreated blood (negative control) (Thabit et al., 2022a).

### 2.6 Elastase assay

The inhibitory impact of sulforaphane on elastase activity at one-fourth of the MIC was evaluated using the Elastin Congo Red (ECR) reagent as previously reported (Ohman et al., 1980; Elfaky et al., 2023). Untreated/sulforaphane-treated (at a concentration of ¼ of the MIC) overnight cultures of PAO1 were adapted to OD_600_ (0.4). Following centrifugation, 0.5 mL of supernatants were combined with the ECR and kept for 8 h at 37°C. Following the removal of insoluble ECR using centrifugation, the absorbances were measured at 495 nm.

### 2.7 Pyocyanin quantification

The impact of sulforaphane at one-fourth of the MIC on the production of pyocyanin was assessed (Cavalu et al., 2022). The fresh cultures of PAO1 in TSB were diluted to OD_600_ (0.4). A volume of 10 μL of the suspensions was introduced into 1 mL of LB broth, either provided or not, with ¼ MIC of sulforaphane. After being incubated for 48 h at a temperature of 37°C and then centrifuged, the optical density was detected at 691 nm.

### 2.8 Withstand to oxidative conditions

The cup diffusion methodology was employed to assess the influence of sulforaphane at a concentration of ¼ MIC on resistance to oxidative stress (Hassett et al., 1995; Hegazy et al., 2020). A freshly prepared culture of PAO1 (50 µL) was evenly distributed on the surfaces of trypticase soya agar (TSA) plates, with or without the addition of sulforaphane at a concentration of ¼ of the MIC. Subsequently, cups were created on plates and subsequently loaded with 30 µL of H_2_O_2_ solution at a concentration of 1.5%. The diameter of the inhibitory zones was measured following 18 h of aerobic incubation at 37°C.

### 2.9 Motility inhibition

To assess the influence of sulforaphane on swarming motility, we used swarming LB agar plates with agar (0.5%) that contained either sulforaphane (at a concentration of ¼ of the MIC) or control plates. Overnight cultures of PAO1 in tryptone broth were diluted, and 2 µL were used to inoculate the surfaces of the plates and then incubated for 18 h. The bacterial motility zones were measured and subsequently compared for analysis (Khayat et al., 2023a).

### 2.10 Virtual affinity to various Lux-type QS receptors

The molecular docking analysis involved the examination of the interaction between sulforaphane and LuxR-type QS systems transcription factors from *Agrobacterium tumefaciens* (Transcriptional activator protein traR; PDB ID: 1L3L), *Pseudomonas aeruginosa* (Quorum Sensing Control Repressor, QscR; PDB ID: 3SZT), and *Chromobacterium violaceum* (CviR transcriptional regulator; PDB ID: 3QP5), along with their co-crystallized ligands, in order to explore their binding modes towards these transcription factors in terms of binding free energies and ligand-receptor interactions. The docking process specifications were applied as previously described (Almalki et al., 2022b). Briefly, the structure of sulforaphane was sketched using ChemDraw Professional software and saved in MDL Molfile (^*^.mol) chemical file format. Then, the saved file was opened in a protein-ligand docking software and prepared for the docking process. The crystal structures of the inspected receptors, together with their ligands, were acquired from the Protein Data Bank (https://www.rcsb.org). Protein preparation for docking analysis was performed following the standard protein preparation procedure integrated into the molecular docking program. H_2_O molecules were eliminated from the retrieved protein, and the protein’s errors were corrected. Protons were added to the protein’s structure and sulforaphane (protonation), then energy minimization was applied. The binding pocket of the protein was defined as all residues within a 10 Å radius of the co-crystallized ligand. The “Docking” module was run to start the docking process. Upon completion of each docking process, 25 docked structures (poses) were generated per ligand, and the ideal pose (the best-docked structure) was chosen according to the lowest energy of the protein-ligand interactions, the binding mode compared to the co-crystallized ligand, and RMSD value as well. The “Ligand Interactions” tool was used to visualize the ligand-protein interactions. The co-crystallized ligand of each retrieved receptor’s crystal structure was re-docked into the investigated receptor in order to validate the docking protocol.

### 2.11 Molecular dynamics simulations

Molecular dynamics (MD) simulations were employed to ascertain the stability of the ligand-receptor complex upon sulforaphane binding to the receptor’s binding site. The MD simulations were executed utilizing the Nosé-Poincaré-Andersen (NPA) equations over 600 picoseconds (ps) duration. The complex system was subjected to a temperature of 300 K (K). Subsequently, a cooling phase was implemented in order to attain the intricate configuration with the minimum energy. With the ultimate goal of exploring the dynamic stability of the complexes formed between the studied quorum-sensing receptors and sulforaphane, the time-dependent potential energy of the complexes was computed throughout MD trajectories. An initial equilibration stage was executed for 100 ps. This phase was executed to facilitate the thermal treatment of the compound, elevating its temperature to 300 K. Subsequently, a production stage of 600 ps was conducted on the complex formed by the ligand and receptor in order to evaluate its stability. The potential energy value was observed over a range from 350 ps to 600 ps.

### 2.12 Expression of QS involved genes

The RNA was extracted from both sulforaphane-treated and control PAO1 strains utilizing the GeneJET RNA Purification Kit (Thermo Fisher Scientific) following the directions provided by the manufacturer. PAO1 was cultivated in LB broth, either with or without sulforaphane at a sub-MIC concentration, and incubated overnight at a temperature of 37°C. After incubation, the suspensions were centrifuged at 12,000 × g for a duration of 5 min in order to gather the cell pellets, which were subsequently suspended in Tris–EDTA buffer supplemented with lysozyme (100 µL) and subjected to incubation at 25°C for 5 min. Afterward, cells were treated with lysis buffer containing β-mercaptoethanol, and the cell lysate was mixed with ethanol and introduced onto a purification column. Contaminants were eliminated by rinsing the column with wash buffers, and the purified RNA was eluted in nuclease-free water. The extracted RNA was preserved at −70°C until further use. To assess the impact of sulforaphane (at ¼ MIC) on the expression of genes involved in the regulation of QS in the PAO1 strain, RNA was purified from bacteria that were either treated with sulforaphane or left untreated and then kept at −80°C (Hegazy and Henaway, 2015; Askoura et al., 2022). The cDNA was produced utilizing the High Capacity cDNA Reverse Transcription Kit from Applied Biosystems. qPCRs were carried out utilizing the SYBR Green PCR Master Mix manufactured by Applied Biosystems, along with the StepOne Real-Time PCR System manufactured by Applied Biosystems. The relative expression of the examined genes to *ropD* (housekeeping gene) was quantified using the method of comparative Ct (∆∆Ct). The primers employed in the qPCRs were previously cited (Khayat et al., 2022a; Khayat et al., 2022b; Khayat et al., 2022c) and listed in Table 1.

**TABLE 1:** qPCR primers used for quantifying the expression of QS genes.

Gene	Primer sequence
*ropD*	F:5′-CGAACTGCTTGCCGACTT-3′
	R: 5′-GCG​AGA​GCC​TCA​AGG​ATA​C-3′
*lasI*	F:5′-CGCACATCTGGGAACTCA-3′
	R: 5′-CGG​CAC​GGA​TCA​TCA​TCT-3′
*lasR*	F:5′- CTG​TGG​ATG​CTC​AAG​GAC​TAC-3′
	R: 5′- AAC​TGG​TCT​TGC​CGA​TGG-3′
*rhlI*	F:5′- GTA​GCG​GGT​TTG​CGG​ATG-3′
	R: 5′- CGG​CAT​CAG​GTC​TTC​ATC​G-3′
*rhlR*	F:5′- GCC​AGC​GTC​TTG​TTC​GG-3′
	R: 5′- CGG​TCT​GCC​TGA​GCC​ATC-3′
*pqsA*	F:5′- GAC​CGG​CTG​TAT​TCG​ATT​C-3′
	R: 5′- GCT​GAA​CCA​GGG​AAA​GAA​C-3′
*pqsR*	F:5′- CTG​ATC​TGC​CGG​TAA​TTG​G-3′
	R: 5′- ATC​GAC​GAG​GAA​CTG​AAG​A-3′

F, forward; R, reverse.

### 2.13 Combination of sulforaphane at sub-MIC with different anti-pseudomonal antibiotics

The impact of sulforaphane at one-fourth of the minimum inhibitory concentration in combination with the chosen antibiotics was evaluated using the checkerboard method, which was employed after determining the MICs of antibiotics against PAO1 by the broth dilution method (Koshak et al., 2023). The influence of the combination was evaluated with selected antipseudomonal antibiotics that represent the different classes of antibiotics, namely ciprofloxacin, cefoperazone, amoxicillin/clavulanic acid, imipenem, gentamycin, tetracycline and chloramphenicol. The combination’s efficacy was assessed using the fractional inhibitory concentration (FIC) index (MIC of the antibiotic in combination/MIC of the antibiotic alone + MIC of sulforaphane in combination/MIC of sulforaphane alone). We measured the optical densities at 600 nm following a 24-h incubation period. FIC index values between 0.5 and 4 showed an indifferent effect; FIC index values greater than 4 suggested antagonism, and FIC index values less than 0.5 indicated synergism.

### 2.14 Mice protection

To determine the *in vivo* inhibitory impact of sulforaphane at ¼ MIC on PAO1 virulence, 3-week-old albino mice were given intraperitoneal injections of sulforaphane (dissolved in dimethyl sulfoxide [DMSO] at a concentration of 0.5% v/v), and their liver and kidney tissues were examined histopathologically (Saqr et al., 2021; Khayat et al., 2023a). There were five groups with five mice each. The first group had PAO1 (1 × 10^6^ CFU/mL) treated with sulforaphane. PAO1 that was left untreated or had been treated with DMSO was injected into the second and third positive control groups. The negative control groups (4th and 5th groups) were left uninfected or injected with sterile PBS. The mice were euthanized after 7 days of observation. Their livers and kidneys were taken out to be homogenized. The bacterial loads were accurately counted by serial dilution in PBS before being plated on Mueller–Hinton (MH) agar plates. The bacterial loads in the organs of mice were assessed using Cetrimide Agar, and expressed as colony-forming units (CFU) per gram of tissue. In addition, the kidneys and liver were extracted from mice, rinsed with normal saline, and subsequently fixed in 10% neutral buffered formalin for histological examination. The samples were dehydrated using increasingly concentrated ethyl alcohol, then cleaned in xylene, infiltrated, and finally embedded in paraffin wax. A rotary microtome was employed to produce slices that were 5 μm in thickness. These sections were then stained with hematoxylin and eosin (H&E × 200) and examined using a light microscope.

### 2.15 Statistical evaluations

The data reported are mean ± standard error in comparison to untreated controls. Unless otherwise indicated, the paired Student's t-test was used to assess significance (*p* < 0.05).

## 3 Results

### 3.1 Sulforaphane inhibited PAO1 growth at low concentration

**FIGURE 1:**
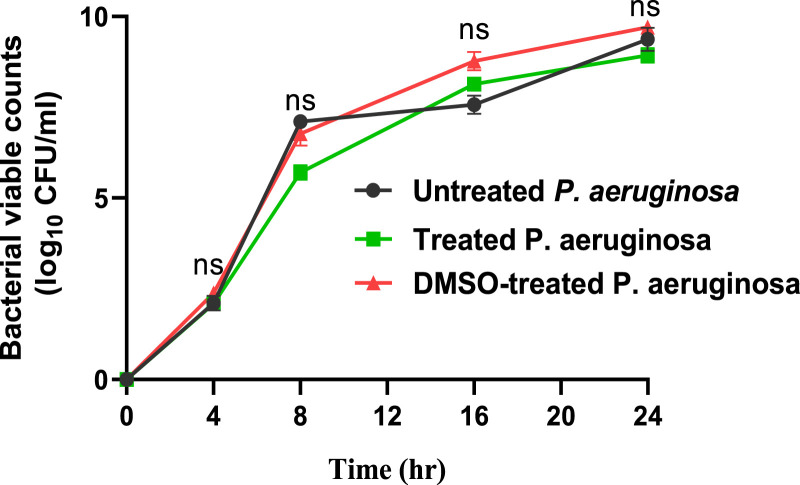
Sulforaphane, when used at a concentration below the minimum inhibitory concentration of 2.5 μg/mL, does not have a noticeable impact on the proliferation of PAO1. A one-way ANOVA test was performed to determine the statistical significance. ns, non-significant.

At 10 µg/mL, sulforaphane inhibited PAO1 growth. Anti-virulence drugs are used at sub-MIC concentrations with the intention of preventing any influence on bacterial growth. Sulforaphane’s anti-virulence properties were assessed in this particular context at ¼ MIC, or 2.5 μg/mL. Viable counts for PAO1 cultured with or without sulforaphane at sub-MIC concentrations were performed to provide additional assurance (Figure 1). Sulforaphane had no discernible effect on the growth of PAO1. To assess the statistical significance, a one-way ANOVA test was applied.

### 3.2 Sulforaphane inhibited the formation of biofilms

**FIGURE 2:**
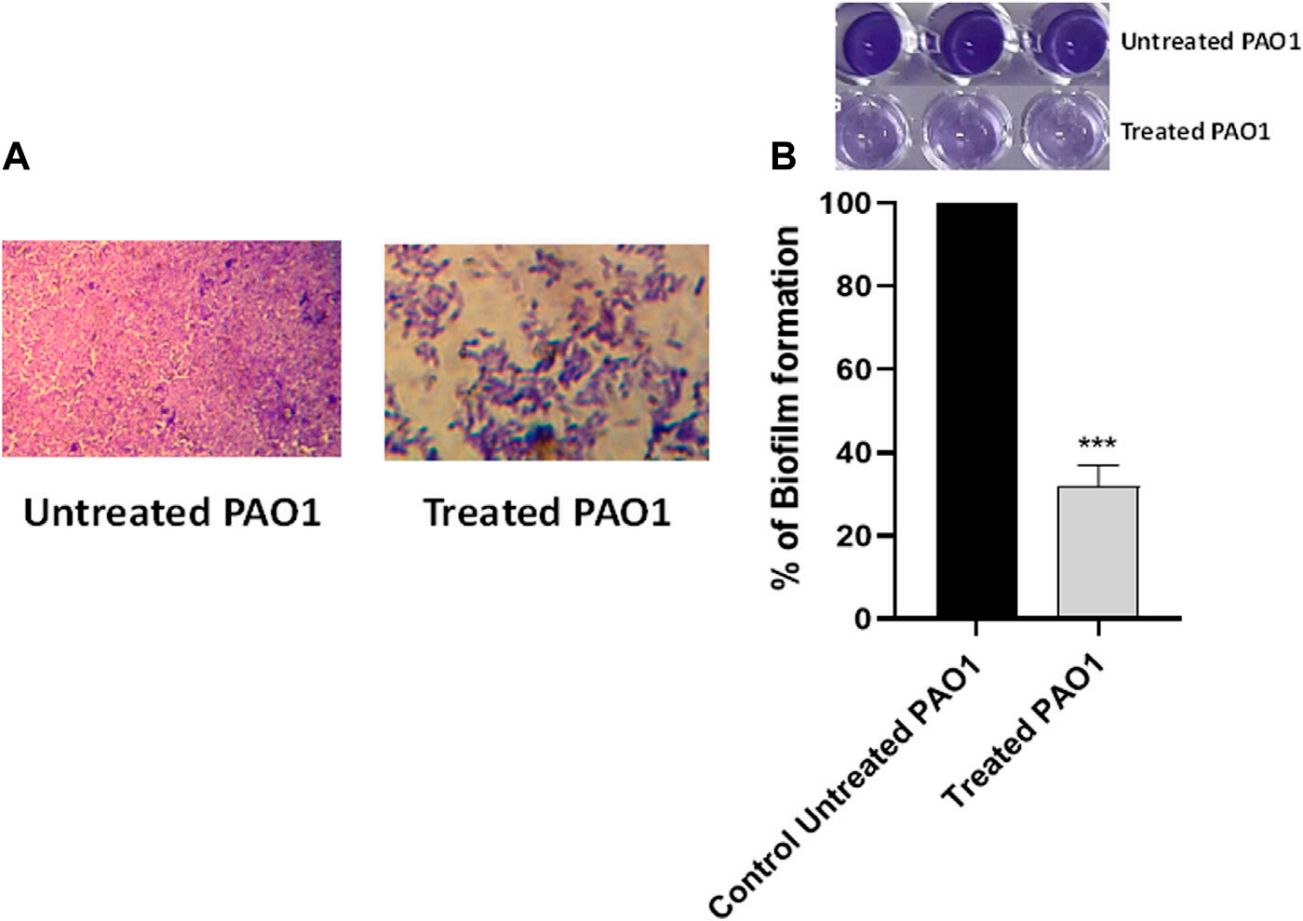
Sulforaphane reduced the production of biofilms in PAO1. **(A)** Photographs depicting the biofilms that were developed with and without the presence of sulforaphane at sub-minimum inhibitory concentration (sub-MIC). **(B)** The optical density of crystal violet, which stained the bacterial cells creating the biofilm, was measured, and the impact on biofilm formation was determined by calculating the percentage change compared to the untreated control. Sulforaphane, when used at a concentration below the minimum inhibitory concentration of 2.5 μg/mL, showed a highly significant (***) reduction in biofilm development.

The crystal violet method was employed to evaluate the inhibitory effect of sulforaphane at a concentration equal to one-fourth of the minimum inhibitory concentration on the production of PAO1 biofilm. Light microscopy images were captured to observe the biofilms that developed on glass coverslips under two conditions: without sulforaphane and with sulforaphane at a concentration of one-fourth of the MIC (Figure 2A). In addition, the optical density of crystal violet, which stained the bacterial cells creating the biofilm, was measured, and the impact on biofilm formation was determined by calculating the percentage change compared to the untreated control (Figure 2B). The results suggest that sulforaphane had a substantial impact on reducing the production of biofilm.

### 3.3 Sulforaphane markedly decreased the synthesis of PAO1 virulence factors and diminished its resistance to oxidative conditions

#### 3.3.1 Effect on protease

**FIGURE 3:**
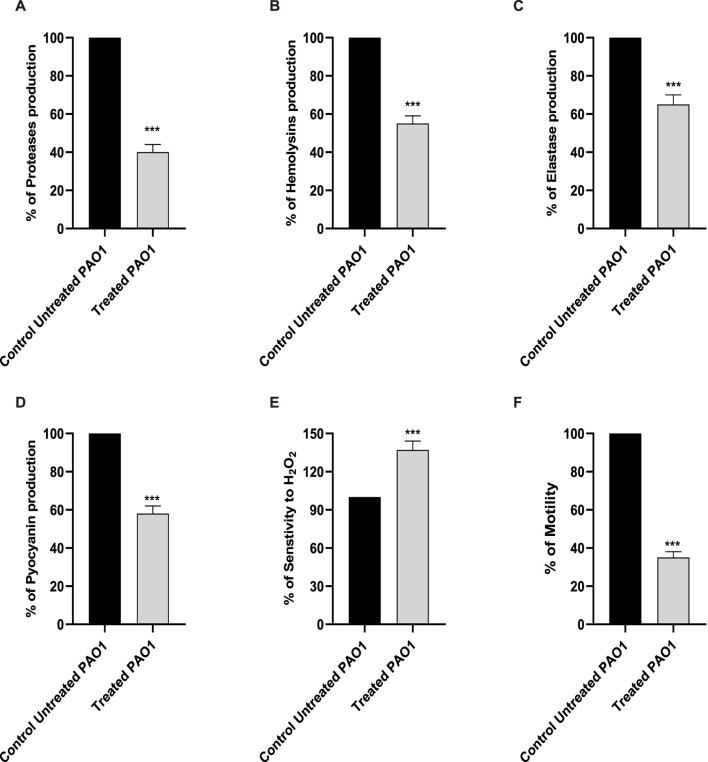
The presence of sulforaphane at sub-MIC levels led to a considerable reduction in the synthesis of PAO1 virulence factors **(A)** proteases, **(B)** hemolysins, **(C)** elastases, and **(D)** pyocyanin. **(E)** Sulforaphane significantly increased the sensitivity of PAO1 to oxidative conditions. **(F)** Sulforaphane, when used at a concentration below the minimum inhibitory concentration (sub-MIC), dramatically reduced the motility of PAO1. The data are displayed as the percentage difference compared to the untreated control. *** indicates a highly significant difference.

Proteases have a significant impact on the pathogenicity of *P. aeruginosa*. The synthesis of proteases was reduced when sulforaphane was present at a concentration that was one-fourth of the minimum inhibitory concentration (Figure 3A). Sulforaphane markedly reduced protease production by around 60%.

#### 3.3.2 Effect on hemolysins

The impact of sulforaphane at a concentration of one-fourth of the MIC on hemolysins was evaluated using un-hemolyzed and fully hemolyzed blood suspensions as negative and positive controls, respectively (Figure 3B). Sulforaphane markedly reduced hemolysin synthesis by roughly 45%.

#### 3.3.3 Effect on elastase

The Congo red methodology was utilized to evaluate the inhibitory impact of sulforaphane at a concentration of ¼ the minimum inhibitory concentration on the generation of elastase (Figure 3C). Sulforaphane greatly reduced elastase production to around 35%.

#### 3.3.4 Effect on pyocyanin production

The impact of sulforaphane at ¼ MIC on pyocyanin synthesis was measured (Figure 3D). The synthesis of pyocyanin was considerably reduced to roughly 42% by sulforaphane at ¼ MIC.

#### 3.3.5 Withstanding oxidative stress

For the purpose of assessing the effect of sulforaphane at a concentration that was one-fourth of the minimum inhibitory concentration on the resistance of PAO1 to hydrogen peroxide, the cup diffusion methodology was employed (Figure 3E). The zones of inhibition in the TSA plates that were treated with sulforaphane exhibited a considerable increase in comparison to the zones of inhibition in the control plates. Sulforaphane enhanced the susceptibility of PAO1 to oxidative stress by roughly 37%.

#### 3.3.6 Effect on motility

The diameters of the PAO1 swarming zones were dramatically reduced in the LB plates supplemented with ¼ MIC levels of sulforaphane compared to the control plates (Figure 3F).

### 3.4 Anti-QS properties of sulforaphane

#### 3.4.1 Molecular modeling showed considered affinity of sulforaphane to different Lux-type QS receptors

##### 3.4.1.1 Docking analysis

Table 2 presents a thorough overview of the docking data, including information on binding free energy, RMSD values, and the types of interactions.

**TABLE 2:** Molecular docking of sulforaphane with LuxR-type QS systems transcription factors from *A. tumefaciens, P. aeruginosa, and C. violaceum*.

*A. tumefaciens* (transcriptional activator protein TraR; PDB ID: 1L3L)				
Compound	ΔG (kcal/mol)	RMSD (Å)	Interaction/bond type	Distance (Å)
Co-crystallized ligand LAE	−9.7	1.17	TYR53: H-acceptor	2.70
			TRP57: H-acceptor	2.86
			ASP70: H-donor	2.83
			THR129: H-acceptor	2.77
Sulforaphane	−6.87	0.80	ASP70: H-donor	3.38
			ASP70: H-donor	3.54
			THR129: H-acceptor	2.91

*P. aeruginosa* (Quorum Sensing Control Repressor, QscR; PDB ID: 3SZT)				
Compound	ΔG (kcal/mol)	RMSD (Å)	Interaction/bond type	Distance (Å)
Co-crystallized ligand OHN	−10.16	1.33	TYR58: H-acceptor	2.88
			TRP62: H-acceptor	3.18
			ASP75: H-donor	2.83
Sulforaphane	−6.01	1.07	SER38: H-acceptor	2.92
			ASP75: H-donor	3.34
			ASP75: H-donor	2.83

*C. violaceum* (CviR transcriptional regulator; PDB ID: 3QP5)				
Compound	ΔG (kcal/mol)	RMSD (Å)	Interaction/bond type	Distance (Å)
Co-crystallized ligand HLC	−8.29	1.19	VAL75: pi-H	4.38
			TYR80: H-acceptor	2.80
			TRP84: H-acceptor	2.92
			ASP97: H-donor	2.86
			SER155: H-acceptor	2.93
Sulforaphane	−5.52	1.1.24	TYR80: H-acceptor	2.90
			TYR88: H-pi	4.11
			SER155: H-acceptor	3.11

RMSD, root mean square deviation; Å, Angstrom.

**FIGURE 4:**
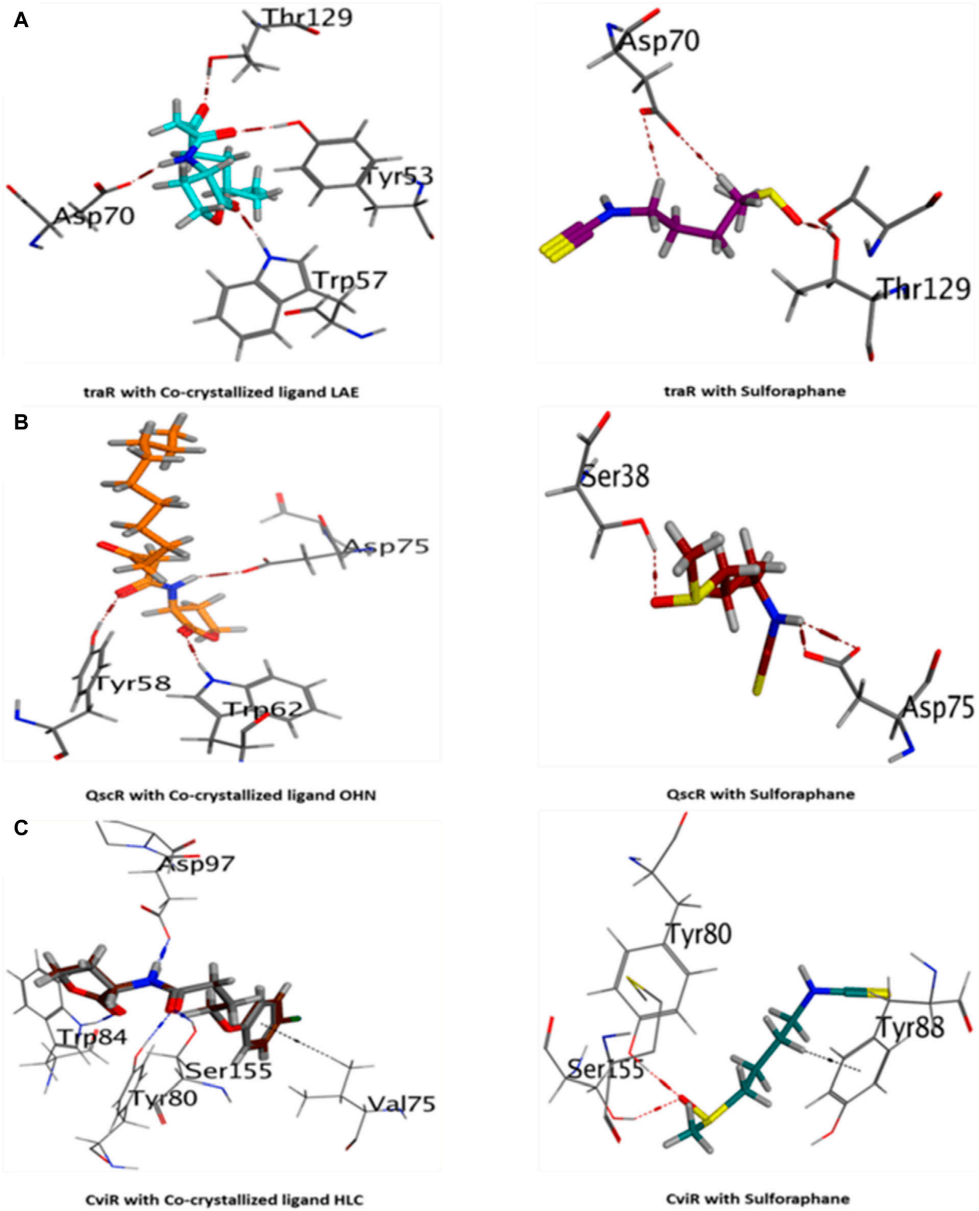
3D visualization of the interactions between sulforaphane and LuxR-type quorum-sensing system transcription factors from *A. tumefaciens* [traR, **(A)**], *P. aeruginosa* [QscR, **(B)**], and *C. violaceum* [CviR, **(C)**].

The current results suggest that sulforaphane has similar interactions to the co-crystallized ligand of TraR, as seen in Figure 4A. Furthermore, ASP75 is crucial in mediating the interaction between sulforaphane and QscR, as well as its co-crystallized ligand (Figure 4B). Noteworthy, Sulforaphane exhibits two amino acid interactions (TYR80 and SER155) that are shared with the co-crystallized ligand of CviR (Figure 4C). The ability of sulforaphane to effectively bind to the receptors TraR, QscR, and CviR is influenced by various factors, such as structural flexibility, conserved binding motifs, hydrophobic and electrostatic interactions, and induced fit mechanisms, despite the differences in amino acid residues present in their binding pockets. The results indicate that sulforaphane has a strong affinity to Lux-type QS receptors that have distinct structures, which could explain why sulforaphane inhibits the generation of virulence factors that QS controls.

##### 3.4.1.2 Molecular dynamics

Both the co-crystallized ligands (i.e., LAE of TraR, OHN of QscR, and HLC of CviR) and the sulforaphane have maintained their binding affinity and remain securely bound to their respective binding sites. Moreover, the potential energy values generated in each complex were distinct.

**FIGURE 5:**
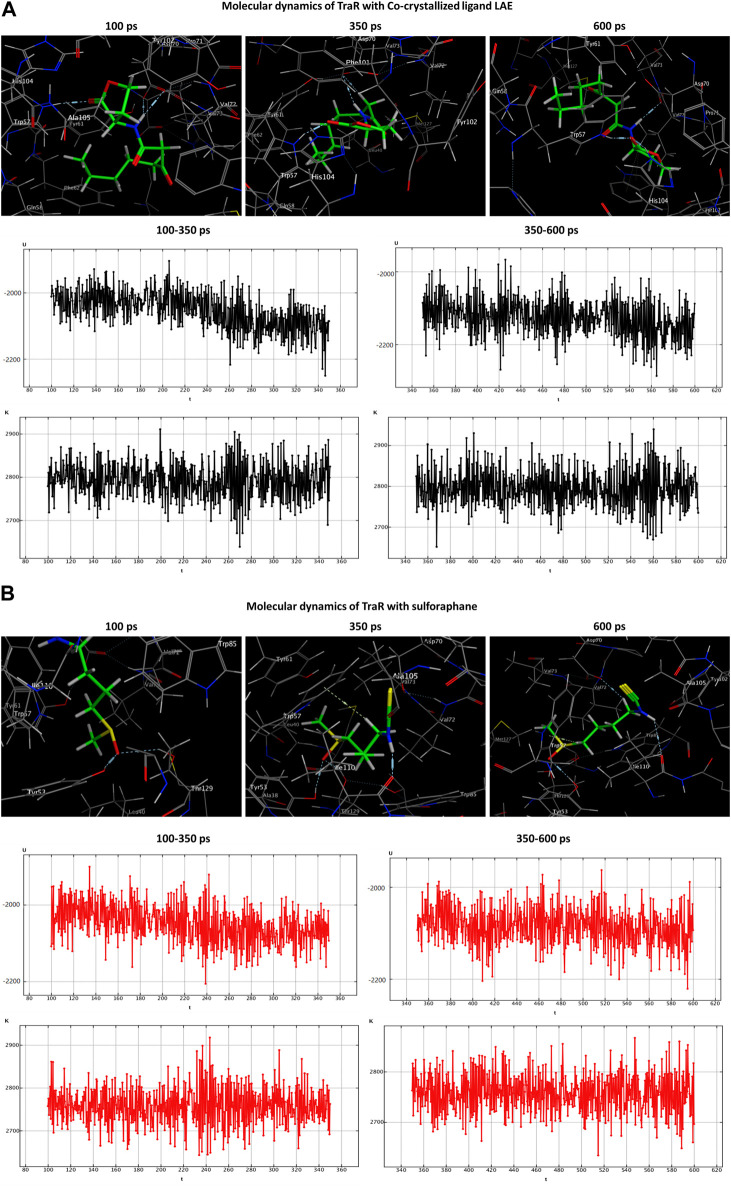
Molecular dynamics simulations of the co-crystallized ligand LAE **(A)** and sulforaphane **(B)** with traR. U, potential energy (kcal/mol); K, kinetic energy (kcal/mol); t, time; ps, picosecond.

During the transition of the LAE-TraR complex from the equilibration phase to the production phase, the ligand engaged in three interactions. Specifically, the ligand formed one hydrogen-acceptor interaction with TRP57 (binding score: −3.0 kcal/mol) and two hydrogen-donor interactions with ASP70 (−0.9 and −1.1 kcal/mol) at 350 ps. The ligand maintained the hydrogen-acceptor and hydrogen-donor interactions with TRP57 (−3.0 kcal/mol) and ASP70 (−3.0 kcal/mol), respectively, until the conclusion of the stage and engaged in a hydrogen-acceptor interaction with HIS104 (−4.3 kcal/mol). During the production phase (at 350 ps) of the sulforaphane-TraR complex, sulforaphane was observed to engage in several interactions, including two hydrogen-acceptor interactions with TYR53 (−2.2 kcal/mol) and THR129 (−3.1 kcal/mol), a hydrogen-donor interaction with ILE110 (−11.1 kcal/mol), a hydrogen-pi interaction with TRP57 (−1.2 kcal/mol), and engaged in an ionic bond with ASP70 (−1.9 kcal/mol). Of note, sulforaphane maintained these bonds until the conclusion of the stage; conversely, the encounter with THR129 was eliminated upon the conclusion of the production phase (Table 3; Figure 5).

**TABLE 3:** Molecular dynamics simulations of sulforaphane with LuxR-type QS systems transcription factors from *A. tumefaciens, P.s aeruginosa, and C. violaceum*.

*A. tumefaciens* (transcriptional activator protein TraR; PDB ID: 1L3L)						
Compound	Interaction type at 100 ps	Interaction energy (kcal/mol)	Interaction type at 350 ps	Interaction energy (kcal/mol)	Interaction type at 600 ps	Interaction energy (kcal/mol)
Co-crystallized ligand LAE	TRP57: H-acceptor	−2.4	TRP57: H-acceptor	−3.0	TRP57: H-acceptor	−3.0
	ASP70: 2 H-donor	−1.3, −3.1	ASP70: 2 H-donor	−0.9, −1.1	ASP70: H-donor	−3.0
					HIS104: H-acceptor	−4.3
Sulforaphane	TYR53: H-acceptor	−4.1	TYR53: H-acceptor	−2.2	TYR53: H-acceptor	−3.5
	THR129: H-acceptor	−1.2	TRP57: H-pi	−1.2	TRP57: H-pi	−0.7
			ASP70: ionic	−1.9	ASP70: H-donor	−1.2
			ILE110: H-donor	−11.1	ASP70: ionic	−4.5
			THR129: H-acceptor	−3.1	ILE110: H-donor	−0.6

*P. aeruginosa* (Quorum Sensing Control Repressor, QscR; PDB ID: 3SZT)						
Compound	Interaction type at 100 ps	Interaction Energy (kcal/mol)	Interaction type at 350 ps	Interaction Energy (kcal/mol)	Interaction type at 600 ps	Interaction Energy (kcal/mol)
Co-crystallized ligand OHN	SER38: H-acceptor	−1.5	TYR58: H-acceptor	−4.0	TYR58: H-acceptor	−1.6
	TYR58: H-acceptor	−1.9	TRP62: H-acceptor	−2.3	TRP62: H-acceptor	−3.7
	ASP75: H-donor	−7.0	ASP75: H-donor	−6.2	ASP75: H-donor	−6.6
			SER129: H-acceptor	−2.1	TRP102: H-pi	−0.6
					SER129: H-acceptor	−1.7
Sulforaphane	SER38: H-acceptor	−3.1	TYR58: H-acceptor	−4.4	TYR58: H-acceptor	−4.4
	TYR58: H-acceptor	−4.3	ASP75: ionic	−0.9	ASP75: ionic	−1.4
	ASP75: H-donor	−10.3	ASP75: ionic	−2.8	ASP75: ionic	−4.2
	ASP75: ionic	−0.6	GLU97: ionic	−3.5	GLU97: H-donor	−15.0
	ASP75: ionic	−1.9	GLU97: ionic	−4.7	GLU97: ionic	−2.5
	MET127: H-donor	−0.9			ASN98: H-acceptor	−0.8

*C. violaceum* (CviR transcriptional regulator; PDB ID: 3QP5)						
Compound	Interaction type at 100 ps	Interaction Energy (kcal/mol)	Interaction type at 350 ps	Interaction Energy (kcal/mol)	Interaction type at 600 ps	Interaction Energy (kcal/mol)
Co-crystallized ligand HLC	TYR80: H-acceptor	−1.5	TYR80: H-acceptor	−2.4	TYR80: H-acceptor	−1.8
	ASP97: H-donor	−7.0	TRP84: H-acceptor	−0.8	ASP97: H-donor	−7.0
	TRP111: H-pi	−1.6	ASP97: H-donor	−7.7	SER155: H-acceptor	−2.2
	SER155: H-acceptor	−2.1	SER155: H-acceptor	−2.5		
Sulforaphane	ASP83: ionic	−4.2	ASP83: ionic	−1.5	ASP83: ionic	−4.3
	TRP84: H-acceptor	−5.4	TRP84: H-acceptor	−2.1	TRP84: H-acceptor	−5.2
			TRP84: H-pi	−0.6		
			GLN87: H-donor	−1.8		
			ASP97: H-donor	−0.6		
			SER155: H-acceptor	−3.3		

ps, picosecond.

**FIGURE 6:**
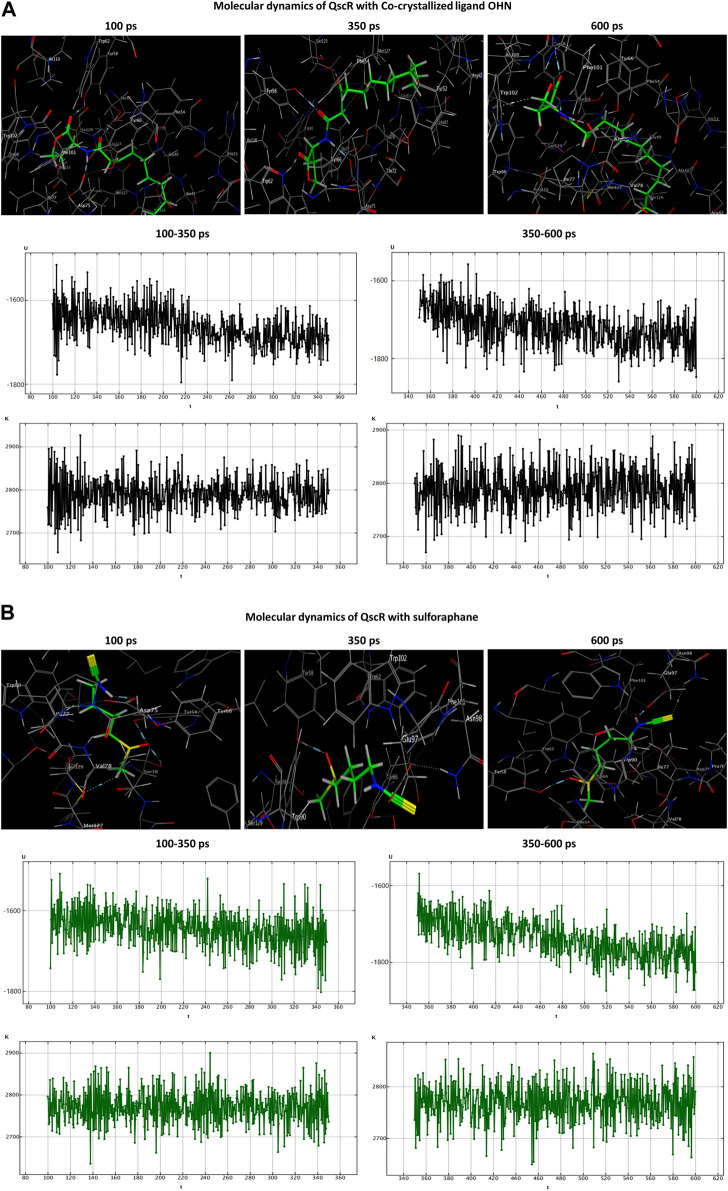
Molecular dynamics simulations of the co-crystallized ligand OHN **(A)** and sulforaphane **(B)** with QscR. U, potential energy (kcal/mol); K, kinetic energy (kcal/mol); t, time; ps, picosecond.

In the OHN-QscR complex, during the production stage (350 ps), the ligand established three hydrogen-acceptor interactions with TYR58 (−4.0 kcal/mol), TRP62 (−2.3 kcal/mol) and SER129 (−2.1 kcal/mol), and a hydrogen-donor interaction with ASP75 (−6.2 kcal/mol), establishing and maintaining these bonds until the conclusion of the stage at 600 ps, with an additional engagement in a hydrogen-pi interaction with TRP102 (−0.6 kcal/mol). On the other hand, sulforaphane in the sulforaphane-QscR complex was observed to engage in several interactions during the production stage, including one hydrogen-acceptor interaction with TYR58 (−4.4 kcal/mol), two ionic bonds with ASP75 (−0.9 and −2.8 kcal/mol), and two ionic interactions with GLU97 (−3.5 and −4.7 kcal/mol). At the conclusion of the production stage, the interactions involving TYR58, ASP75, and GLU97 were maintained. Furthermore, one additional bond involving a hydrogen-acceptor interaction with ASN98 (−0.8 kcal/mol) was manifested (Table 3; Figure 6).

**FIGURE 7:**
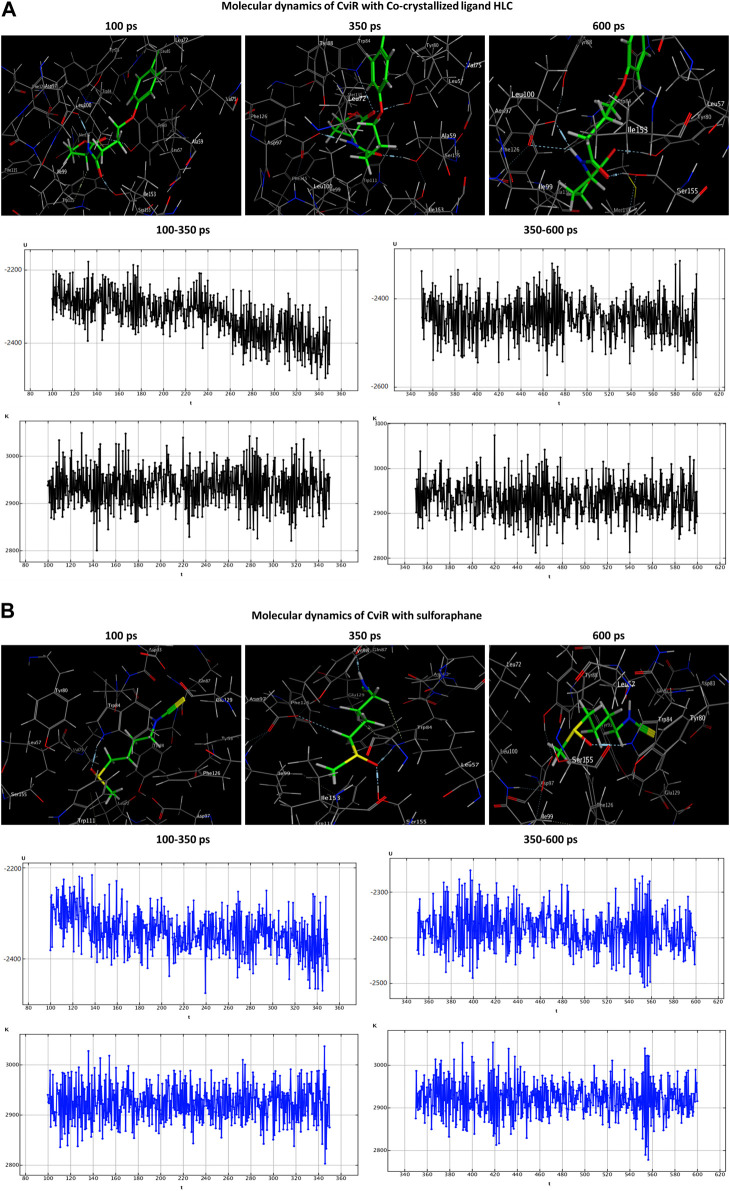
Molecular dynamics simulations of the co-crystallized ligand HLC **(A)** and sulforaphane **(B)** with CviR. U, potential energy (kcal/mol); K, kinetic energy (kcal/mol); t, time; ps, picosecond.

The HLC ligand interacted with CviR through three hydrogen-acceptor interactions with TYR80 (−2.4 kcal/mol), TRP84 (−0.8 kcal/mol) and SER155 (−2.5 kcal/mol), as well as a hydrogen-donor interaction with ASP97 (−7.7 kcal/mol) during the production phase. Notably, at the conclusion of the production stage, the reaction with TRP84 had ceased. During the production phase, sulforaphane, on the other hand, established six interactions with CviR, including two hydrogen-acceptor interactions with TRP84 (−2.1 kcal/mol) and SER155 (−3.3 kcal/mol), two hydrogen-donor interactions with GLN87 (−1.8 kcal/mol) and ASP97 (−0.6 kcal/mol), a hydrogen-pi interaction with TRP84 (−0.6 kcal/mol), and engaged in an ionic bond with ASP83 (−1.5 kcal/mol). Importantly, towards the culmination of the production phase, only two interactions, namely, the ionic bond with ASP83 and the hydrogen-acceptor interaction with TRP84, were maintained (Table 3; Figure 7).

#### 3.4.2 Sulforaphane lessened the expression of QS genes

**FIGURE 8:**
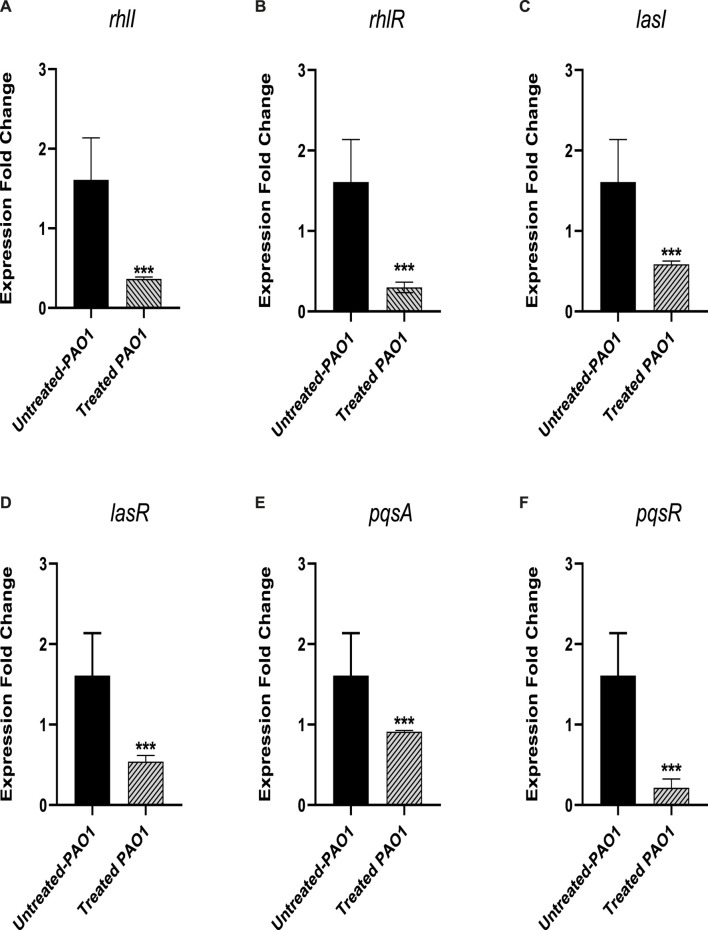
Sulforaphane, at a concentration below the minimum inhibitory concentration (sub-MIC), reduced the expression of the following quorum sensing (QS)-encoding genes in PAO1: **(A)** *rhlI*, **(B)** *rhlR*, **(C)** *lasI*, **(D)** *lasR*, **(E)** *pqsA*, and **(F)** *pqsR*. *** indicates a highly significant difference.

The level of gene expression for the PAO1 QS systems was measured in the presence and absence of sulforaphane at a concentration of 2.5 μg/mL, which is one-fourth of the minimum inhibitory concentration. The expression of all of the QS genes in PAO1 was significantly reduced upon treatment with sulforaphane (Figure 8).

### 3.5 Sulforaphane at sub-MIC protected mice and reduced the PAO1 pathogenesis

**FIGURE 9:**
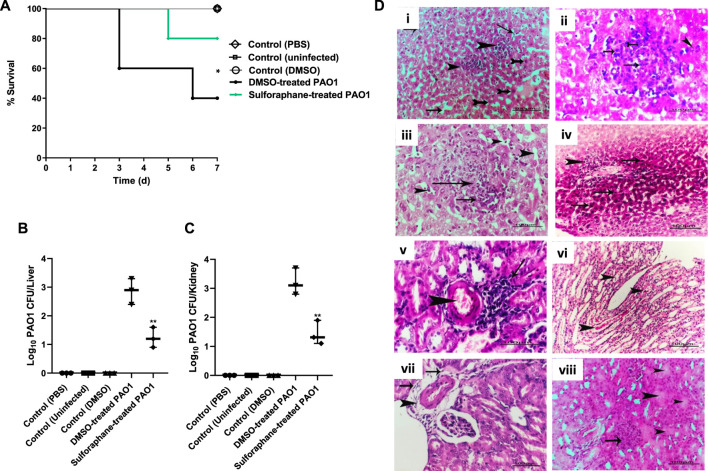
Sulforaphane protected mice and reduced the PAO1 pathogenesis. **(A)** Three fatalities were documented among the mice who received untreated PAO1 injections, but only one fatality was recorded in the group of mice injected with sulforaphane-treated PAO1. No fatalities were documented in the negative control group. The log-rank test for trend was utilized to confirm the statistical significance (*p* = 0.0295). Bacterial loads of liver and kidney tissues in each group are presented in **(B,C)**, respectively. The colonized bacteria in the organ tissues isolated from sulforaphane treated PAO1 injected mice were significantly decreased as compared to tissues isolated from mice injected with untreated PAO1. **(D)** Histopathological organs sections were stained from untreated PAO1 and sulforaphane treated PAO1 using hematoxylin and eosin dye, (i) the liver tissue section of the untreated PAO1 group exhibited substantial and widespread dilation of the hepatic sinusoids (shown by arrows), with focal individualization of some hepatocytes (indicated by tailed arrows) and infiltration of specific areas of the liver parenchyma by leukocytes (indicated by arrowheads), (ii) liver section of infected group showing focal leucocytic cells infiltrated hepatic parenchyma represented by neutrophils (tailed arrow), lymphocytes (arrows) and macrophages (arrowhead), (iii) liver section of sulforaphane treated PAO1 showed dilation of hepatic sinusoids (arrows head), (iv) liver section of sulforaphane treated PAO1 showed perivascular nuclear hyperchromasia of some hepatocytes (arrows) with leucocytic cellular infiltration (arrowhead), (v) kidney section of untreated PAO1 showed focal nephritis of renal cortex represented in congestion of blood vessel (arrowhead)with leucocytic cells infiltration (arrow), (vi) kidney section of untreated PAO1 showed casts deposition inside some renal tubules with the renal medulla (arrowhead), (vii) kidney section of sulforaphane treated PAO1 showed perivascular edema, fibrosis (arrows) with leucocytic cells infiltration (arrowhead) in the renal cortex, (viii) the kidney tissue section of PAO1 treated with sulforaphane exhibited hyper cellularity of renal glomeruli (shown by the arrow), along with degeneration of certain renal tubules (indicated by the arrowhead). (bar = 100 µm). ** indicates a significant difference.

The administration of sulforaphane at a ¼ MIC (2.5 μg/mL) protected mice from PAO1 infection. This was demonstrated by the decrease in the number of deaths, from three in the group that received untreated PAO1 (positive control group) to one death in the group that received treated PAO1 (test group). The Kaplan-Meier estimator was utilized in order to evaluate the mice’s chances of survival, and the log-rank test for trend was utilized in order to ascertain the statistical significance of the findings (*p* = 0.0295) (Figure 9A). As an additional point of interest, the bacterial load in the kidneys and livers of the mice was evaluated for both the group that was injected with PAO1 and the group that served as the control. In order to determine the statistical significance, the one-way analysis of variance (ANOVA) test was carried out. The findings revealed that untreated PAO1 exhibited a much greater degree of colonization in mouse tissues in comparison to PAO1 treated with sulforaphane (Figures 9B, C).

Furthermore, examination of kidney and liver tissues obtained from mice injected with untreated PAO1 demonstrated aberrant tissue pathology characterized by irreversible tissue fibrosis and necrosis. On the other hand, the tissues that were obtained from mice that had been injected with PAO1 and then treated with sulforaphane exhibited modest lesions and mild inflammation. These lesions included congestion of blood vessels and localized leukocyte infiltration (Figure 9D).

### 3.6 The synergistic outcomes of sulforaphane with antibiotics

The minimum inhibitory concentration (MIC) of several antibiotics against the PAO1 strain was assessed. The MIC of combinations of sulforaphane at a quarter of the MIC (2.5 μg/mL) with the tested antibiotics was evaluated. The FIC index was then computed as the result of these combinations (Table 4). It was noted that sulforaphane decreased the MICs of the antibiotics that were evaluated and showed synergistic effects when used together.

**TABLE 4:** The PAO1 strain’s susceptibility to antibiotics in the presence of sub-MIC levels of sulforaphane.

Antibiotic	MICs (µg/mL)	MIC_sulf_ (µg/mL)	FIC index
Ciprofloxacin	4	0.5	0.125
Cefoperazone	128	32	0.25
Amoxicillin/Clavulanic acid	512	128	0.25
Imipenem	8	2	0.25
Gentamycin	16	4	0.25
Tetracycline	512	256	0.5
Chloramphenicol	512	128	0.25

MIC, minimum inhibitory concentration of the examined antibiotics.

MIC_sulf_, Minimum Inhibitory Concentration in the presence of sub-MIC levels of sulforaphane.

FIC index, Fractional Inhibitory Concentration index = MIC of the antibiotic in combination/MIC of the antibiotic alone + MIC of sulforaphane in combination/MIC of sulforaphane alone.

The result of the combination may be antagonistic (FIC index >4), indifferent (FIC index >0.5–4), or synergistic (FIC index ≤0.5).

## 4 Discussion

Resistance to antimicrobial drugs arises due to a variety of processes caused by the selection pressure caused by antibiotic usage (Beceiro et al., 2013; Carattoli, 2013). Over time, bacteria may develop resistance to multiple antibiotic classes, making it more difficult to treat infections and posing a significant public health challenge (Levy, 1992; Agha et al., 2016; Abu Lila et al., 2023; Rajab and Hegazy, 2023). This situation underscores the necessity for innovative strategies and approaches to combat bacterial resistance effectively. In light of the fact that QS plays a pivotal role in regulating the virulence of bacteria, targeting QS pathways could result in the attenuation of bacterial pathogenesis, thereby facilitating bacterial eradication by the immune system. Importantly, due to the fact that they do not directly hinder bacterial growth, such targeting strategies do not put selective pressure on bacteria, which would cause them to evolve resistance (Rasmussen and Givskov, 2006; Jiang and Li, 2013; Hegazy et al., 2022). Although sulforaphane has a low minimum inhibitory concentration of 10 μg/mL, its effects on virulence and QS were assessed at levels below the MIC (1/4 MIC). To ensure confidence in these findings, the impact of sulforaphane at ¼ MIC on PAO1 growth was assessed. The results indicated that it had no significant influence on PAO1 growth.

Quorum sensing (QS) systems are intricate communication networks employed by bacteria to coordinate gene expression in response to alterations in cell density. QS relies on signaling molecules named autoinducers (AIs), which are released by bacteria into their surroundings (Schneider et al., 2002; Rasmussen and Givskov, 2006; Bottomley et al., 2007; Van Houdt et al., 2007; Li and Tian, 2012; Srivastava et al., 2014; Wysoczynski-Horita et al., 2018). In the receptor-inducer model of QS, the existence of AIs in the environment can be detected by bacteria thanks to the presence of specialized receptors (LaSarre and Federle, 2013; Papenfort and Bassler, 2016). Different bacteria utilize different types of AIs and receptors for QS, and the specific mechanisms can vary widely between species. For example, Gram-negative bacteria tend to employ N-acyl homoserine lactones (AHLs) as AIs (Brint and Ohman, 1995; Parsek et al., 1999; Bottomley et al., 2007), and Lux-type QS receptors (Stevens et al., 1994; Janssens et al., 2007; Papenfort and Bassler, 2016; Yu et al., 2020). The Lux-type QS receptors typically contain a characteristic domain known as the LuxR-type DNA-binding domain, which allows them to attach to particular DNA sequences and regulate the expression of genes that are involved in virulence (Papenfort and Bassler, 2016; Almalki et al., 2022b). These receptors often function in conjunction with QS signaling molecules, such as AHLs, which are produced and released by bacteria as they grow. Upon binding to AHLs or other QS signaling molecules, the Lux-type receptors undergo conformational changes that allow them to bind to target DNA sequences known as QS-regulated promoters (Almalki et al., 2022a; Almalki et al., 2022b). This interaction triggers the initiation of the transcription of downstream genes. These genes are tangled in numerous bacterial activities, such as the creation of substances that enable the bacteria to cause disease, the building of biofilms, movement, and resistance to antibiotics (Papenfort and Bassler, 2016). From a structural perspective, there are three primary Lux-type QS systems, namely, *A. tumefaciens* TraR, *P. aeruginosa* QscR, and *C. violaceum* CviR QS receptors (Papenfort and Bassler, 2016). A molecular docking investigation was done to evaluate the affinity of sulforaphane for the three Lux-type QS receptors. The results indicate possible interactions between sulforaphane and these QS receptors. In addition to QscR, *P. aeruginosa* operates three QS systems, including two Lux-type QS receptors, RhLR and LasR, and one additional non-Lux-type receptor, PqsR (Venturi, 2006). A measurement was taken to determine the effect that sulforaphane has on the expression of QS genes in *P. aeruginosa*. The findings demonstrated a notable capability of sulforaphane to decrease the expressions of genes responsible for encoding QS receptors and their associated AIs synthetases. These results propose that sulforaphane has the potential to inhibit QS activity and constrain bacterial virulence. It is worth mentioning that Ganin et al., 2013 demonstrated that sulforaphane effectively inhibits QS in *P. aeruginosa* via its ability to interfere with the generation of the auto-inducers C4-HSL in *P. aeruginosa* (Ganin et al., 2013; Janczewski, 2022). These findings and other reports that attest to the potential anti-QS activities of sulforaphane and related structures (Janczewski, 2022) are in compliance with our results.

Furthermore, the impact of sulforaphane at ¼ MIC on PAO1 biofilm formation was evaluated. Quorum sensing (QS) is responsible for controlling the formation of biofilms and the motility of bacteria, both of which are important virulence factors. Biofilms have a substantial impact on the ability of bacteria to cause disease by providing protection and promoting bacterial persistence and resistance to host defenses and antibiotics (Hoiby et al., 2010; Vestby et al., 2020). Additionally, biofilms facilitate bacterial colonization of host tissues and medical devices, which helps infections to develop and spread (Hoiby et al., 2010; Lila et al., 2023). There is an association between bacterial motility and its capacity to adhere to surfaces, develop biofilms, and spread to initiate infections, as evidenced by the reduced ability of nonmotile bacterial mutants to form biofilms (Hegazy and Abbas, 2017; Askoura et al., 2021). Our findings uncovered the significant antibiofilm effect of sulforaphane at sub-MIC, along with its ability to reduce bacterial swarming. These findings are consistent with Silva et al., who demonstrated that sulforaphane had antibiofilm properties against *Candida species* (Silva et al., 2022) and *Streptococcus mutans* (Zhou et al., 2023).

QS regulation extends to controlling the production of a variety of virulence factors, including hemolysins, elastases, proteases, and other enzymes (Rutherford and Bassler, 2012). Proteases are enzymes that break down proteins, and they contribute to bacterial pathogenesis by degrading host tissues and proteins. This activity helps the bacteria invade tissues and avoid detection by the immune system (Artini et al., 2013). Elastases are enzymes that target elastin, a major component of connective tissue, and their activity leads to tissue damage and inflammation (Galdino et al., 2019). Hemolysins are toxins that disrupt erythrocytes, which ultimately results in the lysis of these cells and the release of hemoglobin. In addition to their role in causing hemolysis, hemolysins can also damage other host cells and tissues, promoting bacterial survival and spread (Strateva and Mitov, 2011). Overall, proteases, elastases, and hemolysins enhance the virulence of bacterial pathogens by promoting tissue damage, inflammation, and immune evasion, thereby facilitating the establishment and spread of bacterial infections (Diard and Hardt, 2017). QS systems allow bacteria to fine-tune their pathogenicity and optimize their interactions with the host environment. Thus, QS represents a key regulatory mechanism that governs the production of these virulence enzymes and contributes to the pathogenesis of *P. aeruginosa* infections (Smith and Iglewski, 2003; Diard and Hardt, 2017). Consistent with sulforaphane’s QS activity, it markedly reduced the formation of elastase, proteases, and hemolysins.

Bacteria have developed various mechanisms to resist oxidative stress (Zhao et al., 2011; Askoura et al., 2016; Askoura et al., 2020). The resistance to oxidative stress contributes to bacterial adaptation and persistence, ultimately facilitating their survival (van Vliet et al., 2002; Guerrero-Beltrán et al., 2012; Bhargava et al., 2014). The blue-green pigment “pyocyanin” is produced by *P. aeruginosa*, and its presence is linked to the severity of pseudomonal infections (Hall et al., 2016). Pyocyanin has been implicated in inflammation, tissue damage, and interference with immune responses. It also plays a role in the development of biofilms.

Additionally, pyocyanin is involved in iron acquisition, a vital element for the growth and survival of bacteria (Jayaseelan et al., 2014; Hall et al., 2016). However, pyocyanin triggers a defensive mechanism in bacteria to withstand oxidative stress and thrive within immune cells (Bhargava et al., 2014). QS-regulated pyocyanin is crucial in inducing oxidative stress within host tissues, resulting in damage to host cells and tissues (Hentzer et al., 2003). Sulforaphane shows promise as an antioxidant agent, effectively reducing oxidative stress and mitigating tissue and cell damage, as reviewed (Guerrero-Beltrán et al., 2012). Besides these anti-oxidant effects, our findings showed the significantly diminishing influence of sulforaphane on both production of pyocyanin and bacterial resistance to oxidative stress. The influence of sulforaphane on reducing the resistance of *P. aureginosa* to oxidative resistance could decrease its capability to survive in the oxidizing conditions in phagosomes that could enhance the immune response for the eradication of invading bacteria. This finding complies with Fahey et al., who showed the lessening effect of sulforpahne on the *H. pylori* intracellular replication (Fahey et al., 2013).

A protection experiment was carried out in mice to determine the anti-virulence impact of sulforaphane against *P. aeruginosa* PAO1. Sulforaphane significantly protected the mice and decreased the PAO1 pathogenesis capacity in mice. Furthermore, sulforaphane reduced the bacterial burden in the liver and kidney and lessened the *P. aeruginosa*-associated inflammation and pathogenesis in liver and kidney tissues. These findings are in compliance with the significant effects of sulforaphane on lessening the generation of virulence factors, as shown above. These *in vitro* and *in vivo* results clearly emphasize the sulforaphane’s anti-virulence activity at low concentrations (2.5 μg/mL), suggesting its possible employment in combination with antibiotics for treating *P. aeruginosa* infections.

Interestingly, the efficacy of sulforaphane in combination with various antibiotics was assessed, revealing significant synergistic effects with all antibiotics tested. Several natural products had substantial inhibitory effects on QS and virulence (Aldawsari et al., 2021; Elfaky et al., 2022a; Khayat et al., 2022c; Khayat et al., 2023b; Koshak et al., 2023); however, sulforaphane acquire more advantage that it is effective at very low concentrations as compared to others. Owing to the pronounced anti-virulence activities of sulforaphane at low doses, it is required to grade up the level of the investigations to employ sulforaphane as an antibiotic adjuvant, which mandates continuing pharmacological, toxicological and pharmaceutical studies to use sulforaphane at proper dose and formulation clinically.

## 5 Conclusion

Sulforaphane is a biologically active compound present in cruciferous vegetables, including cabbage, broccoli, and kale. Our investigation’s results demonstrated sulforaphane’s notable efficacy in reducing the pathogenicity of *P. aeruginosa* at a low dose of 2.5 μg/mL. Sulforaphane significantly decreased the formation of biofilm, movement, and the creation of virulence extracellular enzymes such as proteases, hemolysins, elastase, and other virulence factors, including pyocyanin. In addition, sulforaphane diminished the resistance of *P. aeruginosa* to oxidative stress and mitigated its potential for causing disease in experimental animals. Sulforaphane exhibited synergistic effects by minimizing the MICs of the antipseudomonal pharmaceuticals when taken together. The anti-virulence actions observed may be linked to the downregulation effect of sulforaphane on the genes responsible for QS and its potential ability to impede the receptors involved in QS. In a nutshell, sulforaphane has great potential as an anti-virulence drug, and it can be used alongside conventional antibiotics to manage severe infections effectively. Sulforaphane has the potential to be used as a pharmacophore in the development of more effective anti-QS candidates.

## Data Availability

The raw data supporting the conclusion of this article will be made available by the authors, without undue reservation.
